# Relationships among perceived social support, mindful self-care, and resilience among a sample of nurses in three provinces in China: a cross-sectional study

**DOI:** 10.3389/fpubh.2024.1334699

**Published:** 2024-07-10

**Authors:** Meng Li, Junfan Wei, Shuhua Yang, Yuan Tian, Shan Han, Guanhu Jia, Minerva De Ala, Ruipeng Song, Bo Wei

**Affiliations:** ^1^Nursing Department, The Third People’s Hospital of Henan Province, Zhengzhou, China; ^2^The Seventh Clinical Medicine College of Guangzhou University of Chinese Medicine, Shenzhen, China; ^3^Nursing Department, Beijing Jishuitan Hospital Guizhou Hospital, Guizhou, China; ^4^Nursing Department, The First Affiliated Hospital of Xinxiang Medical University, Xinxiang, China; ^5^School of Nursing Department, Philippine Women's University, Manila, Philippines; ^6^Yellow River Sanmenxia Hospital, Affiliated Hospital of Henan University of Science and Technology, Sanmenxia, China

**Keywords:** mindful self-care, self-care, resilience, nurse, social support, perceived social support

## Abstract

**Objective:**

In this study, we aimed to determine the relationships among perceived social support, mindful self-care, and resilience in a sample of nurses in three provinces of China.

**Methods:**

A cross-sectional study was conducted in seven hospitals in Guangdong, Guizhou, and Henan provinces between August and October, 2023; the provinces are located in south, southwest, and central China. A total of 389 nurses were surveyed using a self-designed sociodemographic characteristics questionnaire and the Chinese versions of Multidimensional Scale of the Perceived Social Support, Brief-Mindful Self-Care Scale and the 10-item Connor-Davidson Resilience Scale (CD-RISC-10).

**Results:**

Of the 389 nurses, the majority were women (*n* = 365; 93.8%), aged 26–35 years (*n* = 244; 62.7%) and had bachelor’s degrees (*n* = 337; 86.6%), had worked for 10–20 years (*n* = 136; 35%), with junior professional titles (*n* = 331; 85.1%), and had a monthly income in the range 3,001–6,000 yuan in CNY (*n* = 239; 61.4%). Nurses’ resilience was measured using the CD-RISC-10, which ranges from 10 to 40 points. And average score of nurses’ resilience was (23.94 ± 6.95). Multiple linear regression showed that scores for resilience were higher among nurses who had higher educational attainment (95% confidence interval: 0.568–3.024, *p* < 0.01), professional titles (95% confidence interval: 0.009–1.693, *p* < 0.05), scores of mindful self-care (95% confidence interval: 0.086–0.155, *p* < 0.01), and scores of perceived social support (95% confidence interval: 0.242–0.328, *p* < 0.01).

**Conclusion:**

Nurses with higher educational attainment and professional titles exhibited higher levels of resilience. Perceived social support and mindfulness self-care are significantly positively correlated with resilience of nurses. The findings of this study are beneficial to further our understanding of nurses’ resilience. The identification of associated factors is conducive to providing more support for nurses who tend to have lower resilience earlier, and can provide useful information for research targeted intervention and support plans aimed at improving nurses’ resilience in the future.

## 1 Introduction

Resilience refers to an individual’s ability to recover from painful experiences in the face of challenges and plays a protective role in managing stress (1). Resilience is essential to handle stress, develop effective coping strategies, and adapt to shifting or rapidly changing circumstances (2, 3). Compared with many other occupations, nurses are more likely to encounter stress (4), such as large number of casualties, shift work, workplace violence and so on (5, 6). The stressors might have a negative impact on the mental health of nurses (7). The consequences of impaired mental health of nurses are extensive, not only affecting the personal relationships of nurses, but also affecting their ability to effectively fulfill their professional responsibilities (8). For example, poor mental health may affect their ability to deliver quality care, pose a potential threat to patients safety and increase the incidents of adverse events (9). In addition, nurses often find themselves at the edge of job burnout in the face of continuous work pressure, and a higher level of resilience is conducive to preventing job burnout (10). A high level of resilience is also conducive to improve job satisfaction (11). Among nurses, especially during the COVID-19 pandemic, higher resilience reduced the incidence of depression, burden of occupational stress (12), and turnover rate (13). Therefore, resilience is particularly important because of its protective effect on nurses’ mental health (14).

Perceived social support refers to the tangible or intangible assistance that an individual receives from friends, families, or society (15). A number of studies have investigated nurses’ perceived social support and resilience. A study conducted under the severe circumstances of the COVID-19 pandemic not only confirmed the positive correlation between social support and resilience, but also emphasized the profound impact of social support and resilience on nurses’ mental health (16). Similarly, a cross-sectional survey (17) conducted among nurses in China found a positive correlation between resilience and perceived social support. Another study (18) conducted in Turkey also confirmed this finding. Encouraging nurses to actively seek social support is often seen by head nurses as a way to develop nurses’ resilience (19).

Mindful self-care is the use of mindfulness for self-care (20). The practice of mindful self-care involves integrating internal and external demands of individuals through mindful thought in order to perform conscious self-care and enhance personal happiness (21). In recent years, a number of interventional studies have been conducted to foster nurses’ resilience using mindfulness methods (22). These interventions were usually based on mindfulness-based stress reduction (MBSR) or extracting some steps from MBSR. Ooms et al. (23) developed a curriculum based on mindfulness training for clinical front-line nurses, which proved effective in improving nurses’ resilience. Furthermore, a survey of palliative care providers during the COVID-19 pandemic also revealed a positive correlation between mindful self-care and resilience (24). However, the relationship between mindful self-care and resilience has not been reported among nurses.

Therefore, we hypothesized that nurses’ resilience was positively correlated with perceived social support and mindful self-care. Because evidence exists to show that the nurse’s perceived social support was positively correlated with resilience (18, 25). And there is a study that show a positive correlation between mindful self-care and resilience among other health care providers (24). In the present study, we investigated nurses for perceived social support, mindful self-care, and resilience. We also investigated factors that could identify nurses with higher levels of resilience.

## 2 Methods

### 2.1 Study design

A cross-sectional (26) exploratory survey method was used in this study because it has been widely used to investigate the status of psychological indicators such as perceived social support (27), mindful self-care (28, 29) and resilience (30). This study has been reported according to the Strengthening the Reporting of Observational Studies in Epidemiology (STROBE) guidelines.

### 2.2 Participants

The Kendall cross-sectional survey sample size estimation formula, *n* = independent variable × (5 ~ 10), was applied to determine the sample size for this investigation (31). Four scales with 16 independent variables were used in this study. The 16 independent variables were derived from the sociodemographic characteristics questionnaire (including six questions), the Chinese Version of the Multidimensional Scale of Perceived Social Support (including three dimensions), the Chinese Version of the Brief-Mindful Self-Care Scale (including six dimensions), and the Chinese version of the 10-item Connor-Davidson Resilience Scale (including one dimension). Assuming that 20% of the responses would be invalid, the minimum sample size required for this study was 100–200.

Participants were recruited with the assistance of head nurses from various institutions. We included nurses who worked in the hospital during the survey period and held regular professional qualification certificates. We also excluded nurses who did not directly work as a nurse in the actual work unit or who had been diagnosed with a mental illness.

### 2.3 Instruments

#### 2.3.1 Sociodemographic characteristics questionnaire

Based on a preliminary study and review, a sociodemographic questionnaire was designed to collect participant characteristics, including gender, age, working years, educational attainment, professional title and average monthly income (in CNY). All of the information collected in this questionnaire was objective.

#### 2.3.2 Chinese version of multidimensional scale of perceived social support

The MSPSS was developed by Zimet et al. (32) in 1988. The MSPSS is a widely validated and reliable instrument used to measure the degree to which an individual feels support from family, friends, and other (33–35). This scale consists of 12 items grouped into three domains, namely family support (four items), friends support (four items), and significant others (four items). The answers range from 1 (strongly disagree) to 7 (strongly agree) on a seven-point Likert scale, with total scores ranging 12–84. Higher total scores on this scale indicate higher levels of perceived social support among participants. The Chinese version of the MSPSS, translated by Jiang et al. (36), demonstrated satisfactory reliability and validity and has been widely employed in diverse studies (37, 38). In the present study, the Cronbach’s α of the scale was 0.971.

#### 2.3.3 Chinese version of brief-mindful self-care scale

This scale is a simplified version of the Mindful Self-care Scale and is used to measure the level of mindful self-care. The English version of the BMSC was developed and validated by Cook-Cottone et al. (37). The Chinese version of the BMSC was translated by Yang et al. (29), which included 24 items covering six dimensions: mindful relaxation, physical care, self-compassion and purpose, supportive relationships, supportive structure, and mindful awareness. Respondents answered on a five-point Likert scale from 1 (never) to 5 (regularly) and total scores ranged 24–120. The higher the total score, the higher the level of mindful self-care. The Chinese version of the BMSC has acceptable validity and reliability in Chinese nurses (29). In the present study, the Cronbach’s α of the scale was 0.943.

#### 2.3.4 Chinese version of 10-item Connor-Davidson resilience scale (CD-RISC-10)

This scale was developed by Campbell-Sills et al. (39) and used to examine the resilience of the participants. The Chinese version of the 10-item CD-RISC-10 was translated by Ye et al. (40) and demonstrated acceptable reliability and validity (40). All items were scored on a five-point Likert scale ranging from 0 (almost never) to 4 (always). The higher the total score, the higher the level of resilience. In the present study, the Cronbach’s α of this scale was 0.965.

### 2.4 Data collection

A cross-sectional survey using convenience sampling was conducted in China. By sending invitations via email, we contacted the directors of nursing departments of several hospitals in Guangdong, Guizhou, and Henan. Some of them expressed interest and agreed to participate in this study. Subsequently, this study was conducted in several hospitals in Guangdong, Guizhou, and Henan between August and October, 2023. The research participants were recruited by the head nurses with the help of the nursing department. Next, the researchers conducted an online training course for the head nurses to teach the way in which the purpose of the study was to be explained to the participants and the process by which the head nurses organized the participants to conduct the survey. The individuals in charge of each hospital personally distributed the paper questionnaires to the participants, who completed questionnaires alone in a quiet conference room. The questionnaires were filled out anonymously, and disclosure of participants’ identity (name, address, etc.) was not involved. After the participants completed the questionnaire, the head nurse checked the questionnaire to make sure there were no missing items. Then, two researchers independently collected the paper questionnaires and input them into a computer; the causes of the differences were identified and resolved in a timely manner. All acquired data is kept on a desktop computer with a password and is backed up on a mobile hard drive to maintain data protection and confidentiality. The researchers involved in this study were the only ones with access to the study data.

### 2.5 Data analysis

The data were analyzed using SPSS Statistics version 24.0 (IBM Corporation, Armonk, NY, United States). Continuous data are presented as mean (standard deviation) and median (interquartile range) for normally distributed and skewed data, respectively. Categorical data are presented as percentages (41). Secondly, in order to explore differences in resilience scores among multiple sociodemographic variables, independent sample t-test (42) or one-way analysis of variance (ANOVA) was used. Pearson correlation analysis was used to determine the correlation between perceived social support, mindful self-care, and resilience. Multi-collinearity was verified by tolerance and variance inflation factor (VIF) (43). When the tolerance is not <0.1 and the VIF is not >10, there is no multi-collinearity. The significant variables in the t-test, ANOVA, and Pearson correlation analysis were considered potential candidates for regression analysis. Finally, multiple linear regression was conducted with potential candidates as independent variables and resilience as the dependent variable to confirm the relationship between nurses’ sociodemographic characteristics, perceived social support, mindful self-care, and resilience. A 2-tailed *p* < 0.05 was considered statistically significant.

### 2.6 Ethical considerations

Ethical approval for this study was obtained from the review board of Third People’s Hospital of Henan Province, Zhengzhou, China (Ethical Review No:2023-SZSYKY-024). The present study was conducted in accordance with the local legislation and institutional requirements. Prior to data collection, the participants were provided with oral and written explanations of the survey and the purpose of the study. The participants then provided their written informed consent to participate in this study.

## 3 Results

### 3.1 Sociodemographic characteristics of participants

In this survey, we invited a total of 400 participants, and finally 389 valid questionnaires were collected, with an effective response rate of 97.25%. Of the 389 participants, the majority were female (365; 93.8%), aged 26–35 years (244; 62.7%), had bachelor’s degrees (337; 86.6%), worked for 10–20 years (136; 35%), with junior professional titles (331; 85.1%), and earned an average monthly income ranging 3,001–6,000 yuan (239; 61.4%). The t-test and ANOVA showed that nurses’ resilience scores were statistically significant in terms of educational attainment and professional title (*P* < 0.05). Specifically, nurses with higher educational attainments and higher professional titles tend to have higher resilience scores. On the contrary, nurses’ resilience did not show statistically significant differences in terms of gender, age, working years, and average monthly income (*p* > 0.05). See Table 1 for details.

**Table 1:** Social-demographic characteristics of nurses and comparison of different variables on resilience (*N* = 389).

Variable	Categories	N	(%)	Mean ± SD	t/F	*p*-value
Gender	Male	24	6.2	25.38 ± 7.93	1.039	0.300
	Female	365	93.8	23.85 ± 6.88		
Age	≤ 25 years old	57	14.7	23.77 ± 5.84	1.593	0.205
	26 ~ 35 years old	244	62.7	23.57 ± 7.21		
	≥ 36 years old	88	22.6	25.10 ± 6.78		
Working years	< 2 years	43	11.1	24.23 ± 7.19	1.531	0.192
	2 ~ 5 years	72	18.5	22.60 ± 5.88		
	6 ~ 10 years	112	28.8	24.52 ± 6.86		
	11 ~ 20 years	136	35	24.67 ± 6.76		
	> 20 years	26	6.7	25.88 ± 6.47		
Educational attainment	Technical secondary school’s degree	2	0.5	20.00	2.759	0.028
	Junior college’s degree	46	11.8	21.50 ± 6.44		
	Bachelor’s degree	337	86.6	24.23 ± 6.97		
	Master’s degree	3	0.8	28.67 ± 0.58		
	Doctor’s degree	1	0.3	35.00		
Professional title	Junior title	331	85.1	23.48 ± 6.71	4.09	0.007
	Intermediate title	33	8.5	25.97 ± 7.91		
	Deputy senior title	24	6.2	27.17 ± 7.46		
	Senior title	1	0.3	35.00		
Average monthly income(in CNY)	≤ 3,000 yuan	125	32.1	23.21 ± 6.96	1.633	0.165
	3,001 ~ 6,000 yuan	239	61.4	24.01 ± 6.94		
	6,001 ~ 9,000 yuan	22	5.7	26.91 ± 6.29		
	9,001 ~ 15,000 yuan	1	0.3	29.00		
	> 15,000 yuan	2	0.5	27.50 ± 10.61		

### 3.2 Perceived social support, mindful self-care, and resilience scores of study participants and their correlations

**Figure 1:**
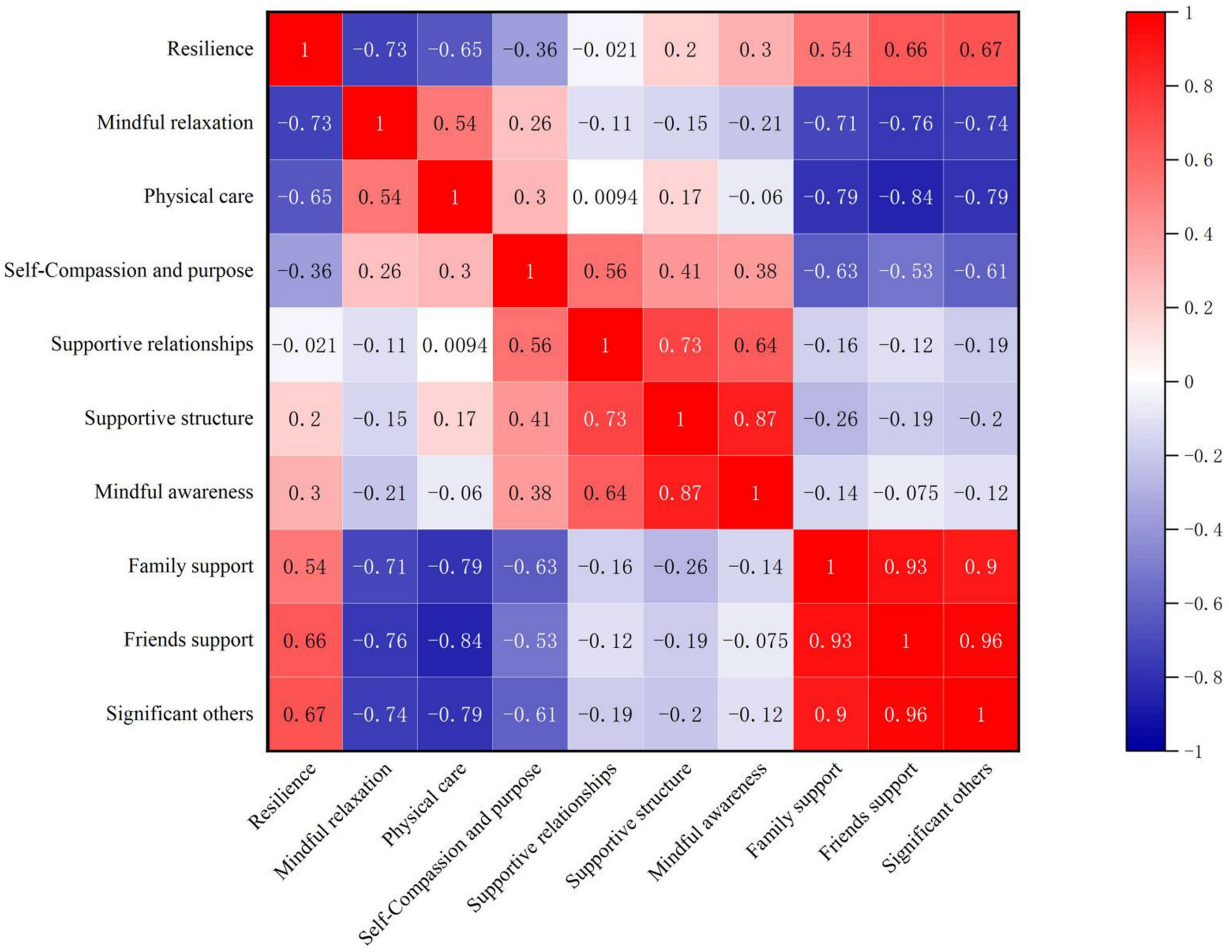
Heatmap of correlations among dimensions of resilience, mindful self-care, and perceived social support.

The overall mean scores of perceived social support, mindful self-care, and resilience were (58.42 ± 12.97), (73.85 ± 16.18), and (23.95 ± 6.95), respectively. Pearson correlation analysis showed that resilience was positively correlated with mindful self-care (*r* = 0.597, *p* < 0.01) and perceived social support (*r* = 0.706, *p* < 0.01). Mindful self-care was positively correlated with perceived social support (*r* = 0.571, *p* < 0.01). See Table 2 for details. See Figure 1 for the correlation heatmap.

**Table 2:** Correlation between nurses’ resilience and the dimensions of mindful self-care and perceived social support.

	Resilience(total score)	Mindful self-care(total score)	Perceived social support(total score)	Mindful relaxation	Physical care	Self-Compassion and purpose	Supportive relationships	Supportive structure	Mindful awareness	Family support	Friends support	Significant others
Resilience (total score)	1	0.597**	0.706**	0.338**	0.357**	0.458**	0.568**	0.649**	0.941**	0.607**	0.691**	0.701**
Mindful self-care (total score)		1	0.571**	0.755**	0.772**	0.816**	0.862**	0.875**	0.571**	0.516**	0.553**	0.548**
Perceived social support (total score)			1	0.360**	0.329**	0.429**	0.575**	0.575**	0.663**	0.929**	0.956**	0.943**
Mindful relaxation				1	0.588**	0.533**	0.490**	0.490**	0.320**	0.334**	0.335**	0.348**
Physical care					1	0.528**	0.543**	0.597**	0.316**	0.302**	0.295**	0.334**
Self-Compassion and purpose						1	0.668**	0.639**	0.451**	0.365**	0.446**	0.404**
Supportive relationships							1	0.846**	0.552**	0.528**	0.562**	0.537**
Supportive structure								1	0.618**	0.506**	0.559**	0.563**
Mindful awareness									1	0.579**	0.651**	0.647**
Family support										1	0.832**	0.792**
Friends support											1	0.878**
Significant others												1

**At the 0.01 level (two-tailed), the correlation was significant.

### 3.3 Multiple linear regression analysis of factors associated with nurses’ resilience

The multi-collinearity test showed that the tolerance of each variable was between 0.670 and 0.961, which was >0.1. The VIF of each variable ranged from 1.041 to 1.491, which were < 10, indicating that there was no multi-collinearity between the variables. Then, variables with statistically significant differences in the t-test, ANOVA, and Pearson correlation analysis were included in the multiple linear regression equation as independent variables. After screening, we included the educational attainment, professional title, mindful self-care, and perceived social support as independent variables in the multiple linear regression equation. According to the multiple linear regression, the educational attainment (95% confidence interval: 0.568–3.024, *p* < 0.01), professional titles (95% confidence interval: 0.009–1.693, *p <* 0.05), scores of mindful self-care (95% confidence interval: 0.086–0.155, *p* < 0.01), and scores of perceived social support (95% confidence interval, 0.242–0.328, *p* < 0.01) were independently associated with nurses’ resilience and explained 56.6% of the variance. See Table 3 for details.

**Table 3:** Multiple linear regression of associated factors of nurses’ resilience.

	B	β	*t*	*p*-value	95%CI	
					Lower	Upper
Constant	−7.806		−3.754	0.000	−1.895	6.282
Educational attainment	1.796	0.098	2.875	0.004	0.568	3.024
Professional title	0.851	0.068	1.988	0.048	0.009	1.693
Perceived social support	0.285	0.533	13.055	0.000	0.242	0.328
Mindful self-care	0.120	0.280	6.859	0.000	0.086	0.155

R = 0.755, R^2^ = 0571, Adjusted R^2^ = 0.566, *p* < 0.001.

## 4 Discussion

In the present study, the average resilience score of nurses was (23.94 ± 6.95), which was lower than that in a previous international study (26.5 ± 7.0) (44). The reason for this difference may be due to the difference in countries and regions where the survey was conducted, or the difference in time when the survey was conducted. However, considering that this study is not a strictly stratified sampling survey based on the entire country, and the participants are not representative of all Chinese nurses, the resilience status of nurses in the entire country is an important issue worthy of further research and discussion.

This study found that nurses with a higher educational attainment were more likely to exhibit higher resilience, which is similar to the results obtained in a study conducted on 1,338 nurses by Ang et al. (45). Another study conducted by Guo et al. (46) also found a strong positive correlation between the highest educational level and resilience score. This may be because the process of obtaining a high educational level is often full of challenges. The higher the level of education, the greater the psychological pressure of students in the learning process (47). Therefore, individuals with higher educational attainment may be more resilient. Another reason may be that individuals with more knowledge may be better at overcoming obstacles. They may be better at using their knowledge to solve bad situations, and may feel more positive, less stressed, and more adaptable to such bad situations. Such individuals (48) may exhibit a more positive attitude in the face of challenges, be less stressed, and ultimately show stronger resilience. That is, individuals with more knowledge are more likely to use that knowledge to adjust their assessments to fit the situation. However, one study (49) showed an opposite conclusion, which found that the resilience scores of nurses who graduated from vocational nursing school is significantly higher than that of nurses with other educational statuses. However, the sample size of this study (49) is small, only 100 nurses participated in this study, of which only 12 nurses graduated from vocational nursing schools, which may lead to a deviation between the results of this study and the real situation. In addition, the difference between our results and the results of this study (49) may also be attributed to the differences in cultural backgrounds and the diversity of sample sources.

The current study also found that individuals with higher professional titles are more likely to have higher levels of resilience than individuals with lower professional titles. This result is similar to those obtained by Ang et al. (45) and Ren et al. (50). The possible reason for these similar results is that nurses with high resilience levels are more likely to calmly face and solve work problems, which helps to improve their career success (51). These individuals may thus be more likely to obtain higher professional titles. As we all know, nurse leaders need to deal with a variety of complex situations (52), with heavy responsibilities and a wide range of management, and have to meet the different needs of others (53). Therefore, high resilience is one of the essential abilities for nurse leaders (54). Highly resilient nurse leadership is conducive to the stability of the team, which in turn affects the retention rate of nurses and the quality of care (55). Given the importance of resilience in shaping the trajectory of the nursing profession, administrators must consider resilience a fundamental factor when evaluating candidates for professional titles. Therefore, nurses with a higher level of resilience are more likely to attain higher professional titles.

The average score of mindful self-care among nurses was (73.85 ± 16.18). Among them, the average scores of mindful relaxation, physical care, self-compassion and purpose, supportive relationships, supportive structure, and mindful awareness were (12.14 ± 3.97), (12.08 ± 3.61), (12.60 ± 3.12), (13.14 ± 3.37), (12.70 ± 3.21), and (10.25 ± 2.23), respectively. These scores are higher than those obtained by Zhang et al. (56) in a survey of 371 oncology nurses in China. In addition, we found that nurses’ mindful self-care was positively correlated with resilience. This result was similar to an existing study result (56). Another survey (24) of palliative care providers during the COVID-19 pandemic found a similar result. This result provides further empirical support for the single-care science resilience model (57), which postulates mindfulness and self-care as strategies for building resilience. Moreover, a study (58) indicated that a steady practice of mindful self-care may be effect in reducing symptoms associated with mental illness, preventing and reducing burnout at work/school, and increase productivity at work/school. This seems to have a certain similarity with resilience, but the specific mechanism through which the two are related needs to be further studied.

Additionally, the average score of nurses’ perceived social support was (58.42 ± 12.97) in this study. This score is slightly lower than those obtained in other surveys (17, 27) conducted in China. This may be due to differences in the geographical areas where the survey studies were conducted. This study also found that perceived social support was associated with resilience. In a previous study, Oksuz et al. (28) conducted a survey of 242 nurses in Turkey, and showed that perceived social support is associated with resilience, similar to the findings of the present study. Similar results were also obtained in another study (16) of 711 registered nurses. This may be due to the fact that support from family or friends can reduce the isolation of nurses in the face of difficulties (59), and the support and help from family and friends provides additional resources to reduce the psychological stress of facing difficulties (60). In addition, other aspects of social support, such as organizational support, may also play an important role in the development of mental resilience in nurses (61).

Although every effort has been made to perfect the design of this study, some limitations are unavoidable. Firstly, this was a cross-sectional study; therefore, causality cannot be inferred. Secondly, we adopted the convenience sampling method, which makes this study less representative although the samples are relatively easy to collect. Thirdly, the number of male nurses in our study was too small, which may lead to a bias in the statistical results regarding whether gender is an influencing factor of nurses’ resilience. In addition, we only identified the influencing factors of nurses’ resilience and did not construct an effective intervention program to enhance nurses’ resilience based on this study, which will be the main goal of our next phase of research. In addition, the geographical location and economic development level of the samples are different, which may have an impact on the psychological state of nurses. Lastly, we took into account that some hospitals may have only 20–30 participants when designing the questionnaire. Considering the study was conducted with the assistance of the nursing directors of each hospital, it may be easy to guess the respondent of a questionnaire in combination with other information once the region of the respondent is known. Therefore, in order to protect the privacy of the participants and ensure the authenticity of the results, we did not collect the information on the participants’ regions. This may result in some potentially important information not being taken into account. In future studies, we should consider improving methods to protect participants’ privacy.

## 5 Conclusion

Educational attainment, professional title, mindful self-care, and perceived social support are positively associated with nurses’ resilience. The findings indicated that nurses with higher educational attainment and professional titles exhibited higher levels of resilience. The findings of this study are beneficial to further our understanding of nurses’ resilience. The identification of associated factors is conducive to providing more support for nurses who tend to have lower resilience earlier, and can provide useful information for research targeted intervention and support plans aimed at improving nurses’ resilience in the future.

## Data availability statement

The original contributions presented in the study are included in the article/supplementary material, further inquiries can be directed to the corresponding authors.

## Ethics statement

Ethical approval for this study was obtained from the review board of Third People’s Hospital of Henan Province, Zhengzhou, China (Ethical Review No:2023-SZSYKY-024). The present study was conducted in accordance with the local legislation and institutional requirements. Prior to data collection, the participants were provided with oral and written explanations of the survey and the purpose of the study. The participants then provided their written informed consent to participate in this study.

## Author contributions

ML: Writing – original draft, Resources, Funding acquisition, Data curation. JW: Writing – review & editing, Writing – original draft, Software, Methodology, Conceptualization. SY: Writing – review & editing, Investigation, Data curation. YT: Writing – review & editing, Investigation, Data curation. SH: Writing – review & editing, Visualization, Supervision. GJ: Writing – review & editing, Visualization, Supervision. MA: Writing – review & editing, Resources, Project administration. RS: Writing – review & editing, Software, Resources. BW: Writing – review & editing, Resources, Methodology, Conceptualization.
